# P04-06 Employing citizen science to promote active and healthy ageing across diverse local urban communities in Birmingham, UK

**DOI:** 10.1093/eurpub/ckac095.060

**Published:** 2022-08-29

**Authors:** Grace Wood, Jessica Pykett, Abby King, Ann Banchoff, Afroditi Stathi

**Affiliations:** 1 School of Sport, Exercise and Rehabilitation Sciences, University of Birmingham, Birmingham, United Kingdom; 2 School of Geography, Earth and Environmental Sciences, University of Birmingham, Birmingham, United Kingdom; 3 Department of Epidemiology & Population Health, Stanford University School of medicine, Stanford, USA

**Keywords:** Older adults, Co-production, Age-friendly, Urban health

## Abstract

**Background:**

Incorporating age-friendly elements across urban environments can promote active and healthy ageing by facilitating opportunities to improve health and well-being among older residents. However, developing inclusive and supportive age-friendly environments remains a key gap for governance and public policy. Community-engaged citizen science recognises older adults as key stakeholders in designing and implementing age-friendly initiatives. The aim of this study was to employ the Our Voice citizen science for healthy equity framework to engage older adults and community stakeholders to: a) identify local urban characteristics that influence active and healthy ageing, and b) co-produce recommendations to develop actionable urban changes.

**Methods:**

Older adults (n = 17; Mean age= 72(7.5 *SD*); 11 women) and community stakeholders (n = 23; 14 women) in urban planning and ageing-well services were recruited from Birmingham, UK. Six online discussion groups (n = 16 older adults, 11 stakeholders), 12 Discovery Tool walks (n = 14 older adults), 3 in-person discussions (n = 12 older adults), 2 online individual discussions (n = 2 older adults) and 2 workshop events (n = 15 older adults, 17 stakeholders) were conducted. Audio transcripts and co-produced data were member checked and thematically analysed to identify urban barrier and facilitator themes and co-produce recommendations.

**Results:**

A range of interconnected urban features were identified as influential of active and healthy ageing, including presence or absence of community facilities, suitable outdoor spaces, and the impact of Covid-19. Six collective and 12 individual recommendations were co-produced proposing feasible ways to enhance urban environments. These included public toilets schemes, maintenance of green and public spaces, car parking enforcement, provision of local information, and integrating communities across all ages.

**Conclusion:**

Employing citizen science developed a network of older adults and stakeholders that shared local knowledge and experiences to co-produce a strong vision for shaping urban environments in Birmingham. This approach facilitated older adults to: drive research processes and solution-building; identify local urban influences; and advocate these findings to a network of actors who can disseminate and activate change in urban domains. To enhance citizen science further, increased time and resources to embed older adults into scientific processes, including data analysis and interpretation, is required.

